# Prevalence of *Salmonella enterica* serovar Typhi infection, its associated factors and antimicrobial susceptibility patterns among febrile patients at Adare general hospital, Hawassa, southern Ethiopia

**DOI:** 10.1186/s12879-020-05726-9

**Published:** 2021-01-07

**Authors:** Roza Nasir Awol, Dawit Yihdego Reda, Deresse Daka Gidebo

**Affiliations:** 1 SNNPR, Hawassa Public Health Laboratory, Hawassa, Ethiopia; 2 School of medical laboratory Science, College of medical and Health Sciences, Hawassa University, Hawassa, Ethiopia

**Keywords:** *S.typhi*, Febrile patient, Hawassa, Ethiopia

## Abstract

**Background:**

*Salmonellas enterica* serovar Typhi (*S.typhi*) causes typhoid fever and is a global health problem, especially in developing countries like Ethiopia. But there is a little information about prevalence and factors association with *S.typhi* and its antimicrobial susceptibility pattern in Ethiopia especially in the study area.

The aim of this study was to determine the prevalence of *S.typhi* infection, its associated factors and antimicrobial susceptibility pattern among patient with a febrile illness at Adare General Hospital, Hawassa, Southern Ethiopia.

**Methods:**

Hospital based cross sectional study was conducted among 422 febrile patients from May 23, 2018 to October 20, 2018. A 5 ml venous blood was collected from each febrile patient. Culture and biochemical test were performed for each isolate. Antimicrobial susceptibility testing was performed for each isolate using modified Kirby-Bauer disk diffusion techniques.

**Result:**

In this study, the prevalence of *S.typhi* among febrile illness patients at Adare General Hospital was 1.6% [95% confidence interval (CI): 0.5–2.9]. The age of the study subjects were ranged from 15 to 65 years (mean age 32 years). It was observed that participants who came from rural area had 8 times (AOR 8.27: 95% CI: 1.33, 51.55) more likely to had *S. typhi* infection when compared with urban dwellers. The microbial susceptibility testing revealed that all six of *S.typhi* isolates showed sensitive to Ceftriaxone and all 6 isolates showed resistant to nalidixic acid and Cefotaxime and 5(83.3%) susceptible to Chloramphenicol and Ciprofloxaciline. Multidrug resistance (resistance to three or more antibiotics) was observed among most of the isolates.

**Conclusion:**

S. typhi bacteraemia is an uncommon but important cause of febrile illness in our study population. Ceftriaxone therapy is a suitable empirical antibiotic for those that are unwell and suspected of having this illness. Further surveillance is required to monitor possible hanging antibiotic resistant patterns in Ethiopia.

**Supplementary Information:**

The online version contains supplementary material available at 10.1186/s12879-020-05726-9.

## Background

*Salmonella* belongs to the family Enterobacteriaceae. Within two species, *Salmonella bongori* and *Salmonella enterica*, over 2500 different serotype or serovar have been identified [1]. The only known natural hosts and reservoir for *S.typhi* infections are low socioeconomic condition, deprived hygiene with human beings [2].. A very comparable but often less sever disease is caused by paratyphoid fever, which is caused by *S.enterica* serovar Paratyphoid A (SPA),B,C [3]. The organisms are non-capsulated, non-sporulating, gram negative, facultative anaerobic bacilli, which have characteristic flagellar, somatic and outer coat antigens [2].

Typhoid fever is a global health problem. It is an acute, life-threatening, febrile illness. Its real impact is difficult to estimate because the clinical picture is confused with those of many other febrile infections [3]. Without treatment, the case fatality rate of typhoid fever is 10–30%, however, an appropriate therapy may decreases the case fatality to 1–4% [4]. Despite the availability of antibiotics and different prevention method, almost 80% of the cases and deaths occur in Asia and the rest occur mostly in Africa and Latin America [5].

In many developing countries, especially in sub-Saharan Africa, the true burden of enteric fever is difficult to estimate due to the limited diagnostic resources and proper surveillance tools result in poor characterization of the burden of enteric fever [6, 7].

The global encumber of disease estimation for typhoid were estimated based on community-based incidence studies using climatic change and socio-economic features to derive continental estimates of the burden. In most African countries the recommended estimate incidence of typhoid were 10–100 cases/100,000 person years in most African countries with the incidence highest in childhood [8]. Because of the limited scope of studies, under-reporting of the case, the presence of other disease and lack of coordinated epidemiological surveillance system in Ethiopia it was difficult to evaluate the burden of typhoid fever infection [9].

In a study conducted in Jiggiga, Ethiopia the overall prevalence of enteric fever was 11%. The prevalence of *S.typhi* (7%) was higher than *S.paratyphi* (4%). The odds of having enteric fever were higher among the study participants aged 31–45 years and with previous history of enteric fever [10].

The risk for infection is high in low- and middle-income countries where typhoidal Salmonella is endemic and that have poor sanitation and lack of access to safe food and water [11] and the peak incidence is reported in children between 5 and 19 years of age in developing countries. But some studies in South Asia report highest rates of enteric fever under 5 years of age [12].

Access to safe water and sanitation is inadequate in many parts of the world. The scarcity of these basic amenities weighs heavily on public health and typhoid fever [13]. Regions with contaminated water supplies and inadequate waste disposal have a high incidence of typhoid fever [14].

Some patients excreting *S.typhi* have no history of typhoid fever which means they do not recollect a recent febrile illness with diahoea. Between 1 and 5% of patients with acute typhoid infection have been reported to become chronic carriers of the infection in the gall bladder, depending on age, sex and treatment regimen. The propensity to become a chronic carrier may have changed with the present availability and selection of antibiotics as well as with the antibiotic resistance of the prevalent strains [3].

In Ethiopia, as in other developing countries, the prevalence and real situation of antibiotic resistance is also not clear since *Salmonella* are not routinely cultured and their resistance to antibiotics cannot be tested. However, to control the spread of typhoid fever, surveillance for *S.typhi* and the assessment of antimicrobial susceptibility is essential [9]. Therefore; the aim of this research is to determine prevalence of *S.typhi* infection and its associated risk factor and its antimicrobial susceptibility pattern among patient with febrile illness.

## Method

### Study area

Hawassa town is the capital city of Southern Nations, Nationalities and Peoples Regional state. The city is located on the shores of Lake Hawassa in the Great Rift Valley and is located 275 km to the South of Addis Ababa.

### Study design and period

Hospital based cross sectional study was conducted from May 23, 2018 to October 20, 2018, at Adare General Hospital, Hawassa, Ethiopia.

### Study population

All febrile patients who fulfilled the inclusion criteria and requested for Widal test at Adare General Hospital, Hawassa, Ethiopia.

### Inclusion criteria

The following patients who fulfilled all of the following criteria were included in the study:

1) Those who were febrile (defined as a temperature of 37 °C).

2) Those aged over 15 years old.

3) Those whom the treating physician suspected may have typhoid fever (demonstrated by the ordering of the “Widal test” with its’ known limitations.)

4) Those able and willing to consent to participation.

### Exclusion criteria

The following patients were excluded from the study:

1) Those unable to consent to participation due to severity of illness.

2) Those who had received antibiotics within the 2 weeks prior to presentation.

3) Those presenting on more than one occasion during the study period had their first attendance included only.

### Sample size determination

Sample size was calculated by using single proportion formula

$$ \mathrm{n}={{\mathrm{Z}}^2}_{\upalpha /2}\ \mathrm{p}\ \left(1-\mathrm{p}\right)/{\mathrm{d}}^2 $$

Where n: sample size.

Z – Standard normal distribution value at the 95% CI, which is 1.96.

P – The prevalence was taken as, 50%.

d – The margin of error, taken as 5%.

Sample size = n (sample size) + (10% non-respondent).

Sample size (N) = 384 + 38.4= 421.4 ~ 422.

Samples collected during the study was 422.

### Sampling technique and procedure

Systematic random sampling method was used to recruit patients attending outpatient department of Adare General Hospital. Considering a five month study period, an estimated of 1320 patients visited the outpatient department according to hospital plan and the past three months performance document review. This estimate was divided by the sample size to determine the sample interval (k value), which would be 3. The 1^st^served patient was selected by lottery method and every 3rd patients thereafter were invited to participate in the study until the required sample size was obtained.

### Data collection

A pre-tested and pre-structured questionnaire was used to collect information on socio-demographic characteristics (age, residence, marital status, and educational level) and associated factors (Additional file 1).

Culture and identification of *S.typhi* About 5 ml of venous blood sample was collected from each febrile patients and the sample was directly inoculated into bottle containing 45 ml triptic soya broth medium (Himedia,India) and incubated for 7 days. Those cultured bottle which showed growth were further sub cultured on MacConky agar (Deben diagnostic Ltd) and blood agar media (Biomark, India laboratories) after 48 h. Negative broth culture were incubated for seven days and sub cultured before reported negative. Suspected colonies obtained were screened by biochemical test using triple sugar iron agar (TSI), citrate utilization test, SIM (sulfide indole motility test), urease test and lysine decarboxylation test. Specific antisera were used to determine *S.typhi.*

### Antimicrobial susceptibility test

Antimicrobial susceptibility test was done for the isolates of *S.typhi* using Muller-Hinton Agar (MHA) (Biomark, India laboratories) following the disk diffusion technique. Each isolate was tested for the selected antimicrobial agent such as ciprofloxacine (5 μg), cotrimoxazole (25 μg), cefotaxime (30 μg), ceftriaxone (5 μg), nalidixic acid (30 μg), chloramphenicol (30 μg) and ampicillin (10 μg) (Abetek biological Ltd). After incubation for 18–24 h at 37^o^c, 4–5 colonies was transferred to a tube containing 5 ml sterile normal saline by using inoculating loop. Turbidity of the broth was matched with 0.5 McFarland standards. A sterile cotton swab was dipped in to the suspension. The swab rotated and pressed firmly against the inside wall of the tube to remove excess inoculum. The swab was streaked on the surface of MHA and antibiotic disk were placed on the plate then the plate were incubated at 35-37^o^c for 18–24 h. The diameter of the zone of inhibition around the disk was measured using a metal caliper and the isolate were classified as sensitive, intermediate and resistant as recommended by CLSI 2018.

### Quality assurance

The English version of the questionnaire has been converted to home language (Amharic) and reverses to English to make sure its uniformity by two individuals who have language professional and medical background (Additional file 1). Earlier to the commencement of data collection, every data collectors were taught by the principal investigator. The collected data were checked every day for reliability and truthfulness. Standard Operating Procedures (SOPs) were rigorously pursued during sample collection, storage and analytical process. A pperformance test of the broth (internal quality control) has done by branded strain of *Escherichia coli* ATCC 25922, *Staphylococcus aureus* ATCC 25923. For quality control of blood agar *Streptococcus pyogenes* ATCC 19604 and *streptococcus pneumonia* ATCC 49619 were used and for MacConkey, *Escherichia coli* ATCC 25922 and *Salmonellatyphimurium* ATCC 13311 were used.

### Data entry and analysis

Data entry, cleaning and analysis was done using SPSS version 23.0 software. First descriptive statistics was computed using frequency and percentage. The bivariate analysis was performed to select candidate variables for multivariate logistic regression analysis. Variables with *p*-value < 0.25 on bivariate analysis were selected for multivariate analysis. The final model was used to determine the association between explanatory variables and the outcome variables.

## Result

### Socio-demographic characteristics

A total of 422 study participant were enrolled in this study with an overall response rate 381 (90.28%). Of these, 172(45.1%) were males and 209(54.9%) were females. The mean age was 32 years and standard deviation (SD), 11.64; range, 15–65 years. Nearly, one-third of the study participants 136 (35.7%) were in the age category of 15–24 years. The majority of the study participants were urban 324 (85.3%) residence. Concerning the marital status 223(58.5%) were married and 208(54.6%) had completed secondary school and above (Table 1).

**Table 1:** Socio-demographic characteristics and its distribution of *S.typhi* of febrile patients visiting outpatient department of Adare General Hospital, from May 23/2018 to October 20/2018 Hawassa, Ethiopia

Variables	Number of tested (%)	Number of positive for ***S.typhi*** (%)
**Sex**		
Male	172 (45.1)	2 (1.2)
Female	209 (54.9)	4 (1.9)
**Age (in years)**		
15–24	136 (35.7)	1 (0.7)
25–34	114 (29.9)	3 (2.6)
35–44	74 (19.4)	1 (1.4)
45 and above	57 (15.0)	1 (1.8)
**Residence**		
Urban	324 (85.0)	2 (0.6)
Rural	57 (15.0)	4 (7.0)
**Marital Status**		
Married	223 (58.5)	2 (0.9)
Single	144 (37.8)	3 (2.1)
Widowed and Divorced	14 (3.7)	1 (7.1)
**Educational Status**		
No formal education	30 (7.9)	1 (3.3)
Primary education	143 (37.5)	3 (2.1)
Secondary and above	208 (54.6)	2 (1.0)
**Occupation**		
Employed	93 (24.4)	1 (1.1)
Merchant	140 (36.7)	2 (1.4)
Student	79 (20.7)	2 (2.5)
Housewife	63 (16.5)	1 (1.6)
Other	6 (1.6)	0 (0.0)
**Family size**		
< 3	194 (50.9)	3 (1.5)
4–6	180 (47.2)	3 (1.7)
> 7	7 (1.8)	0 (0.0)

From the total study participants 367(96.3%) use tap water for drinking and 14(3.7%) uses river water, wall water or unprotected spring water for drinking purpose. Sixty one (16%) of the participants were treating water before drinking while 320(84%) were not use any treatment for drinking water (Table 2).

**Table 2:** Hygienic practice and *S.typhi* distribution among febrile patients visiting outpatient department of Adare General Hospital, from May 23/2018 to October 20/2018 Hawassa, Ethiopia

Categories	Number of tested (%)	Number of positive for ***S.typhi*** (%)
**Hand washing practice after latrine**		
Yes	249 (65.4)	1 (0.4)
No	132 (34.6)	5 (3.8)
**Use of soap for hand washing**		
Always	97 (39.0)	0 (0.0)
Some times	100 (40.2)	0 (0.0)
Never	52 (20.9)	1 (1.9)
**Time of hand washing**		
After meal	153 (40.2)	3 (2.0)
Before and after meal	228 (59.8)	3 (1.3)
**Eating meal at**		
Hotel	55 (14.4)	1 (1.8)
Home	119 (31.2)	2 (1.7)
Home and hotel	207 (54.3)	3 (1.4)
**Eating of food from street vender**		
Yes	189 (49.6)	3 (1.6)
No	192 (50.4)	3 (1.6)
**Washing of vegetables or fruits before eating**		
Yes	228 (59.8)	2 (0.9)
No	153 (40.2)	4 (2.6)
**Where do you get drinking water**		
River	5 (1.3)	0 (0.0)
well water	8 (2.1)	0 (0.0)
unprotected spring water	1 (3)	0 (0.0)
tap water	267 (96.3)	6 (2.2)
**Treating drinking water**		
Yes	61 (16)	0 (0.0)
No	320 (84)	6 (1.90)
**Washing of hands before preparing food**		
Always	132 (34.6)	0 (0.0)
Sometimes	183 (48.1**)**	6 (3.3)
No	66 (17.3)	0 (0.0)
**Recent infection with typhoid fever in the family members**		
Yes	44 (11.5)	0 (0.0)
No	337 (88.5)	6 (1.8)
**Have you ever suffered from typhoid fever**		
Yes	84 (22)	0 (0.0)
No	297 (78)	6 (2)

The availability of latrine among study participants were 379 (99.5%). The remaining had no latrine service. The hand washing habit of the study participants after latrine were 249(65.4%). Among them 97(39%) were using soap always, 100(40.2%) were sometimes and 52(20.9%) were not at all. From the total were respond latrine available in their home, while 2(0.5%) were respond no latrine in their home. From the total study subject were washes their hands after latrine. Among the study subject who were washes their hands, uses to washes their hands but uses soaps sometimes washing their hands after latrine, the rest were not use soap or only washes their hands with water (Table 2).

### Prevalence of *S.typhi*

In this study, the overall prevalence of *S.typhi* were 1.6%.Additionally *Staphylococcus aureus* 25(6.56%). coagulase negative *Staphylococci* 20 (5.2%), *E.coli* 4(1%), *Proteus* spp. 2(0.5%), *Enterococci* 3(0.8%), *Streptococci* 3(0.8%), *Klebseilla* spp. 1(0.3%) and *Pseudomonas* spp. 1(0.3%) were observed.

The highest prevalence of *S.typhi* infection was observed among patients in the age group of 25–34 years old was 3(2.6%). Based on marital status, 1(7.1%) widowed and divorced patients were positive for *S.typhi* infection. With regard to site of residence, 7% of patients came from rural area were positive for *S.typhi* infection. Those patients with no formal education was 1(3.3%) for *S.typhi* and 2(2.5%) the students were positive for *S.typhi* (Table 1). The highest frequency of S.typhi infection were observed patient who were not washing hands after latrine 5(3.8%). (Table 2).

### Associated factors for *S.typhi* infection

In the bivariate analysis, age, residence, marital status, hand washing practice, washing of vegetables or fruits before eating were candidate variable for multivariable analysis (Table 3).

**Table 3:** Associated factor of *S.typhi* infection among febrile patients attending Adare General Hospital, from May to October 2018,(N-=381)

Variables	S. typhi					
	Number of tested (%)	Number of positive (%)	COR (95% CI)	P-value	AOR(95% CI)	P-Value
**Age (in years)**						
15–24	136 (35.7)	1 (0.7)	1			
25–34	114 (29.9)	3 (2.6)	3.65 (0.37,35.57)	0.250*	2.76 (0.19,39.26)	0.453
35–44	74 (19.4)	1 (1.4)	1.85 (0.11,30.00)	0.665	1.64 (0.09,31.22)	0.744
45 and above	57 (15.0)	1 (1.8)	2.41 (0.15,39.22)	0.536	4.93 (0.25,97.51)	0.295
**Residence**						
Urban	324 (85.0)	2 (0.6)	1			
Rural	57 (15.0)	4 (7.0)	12.15 (2.17,67.99	0.004**	8.27 (1.33,51.55)	0.024*
**Marital Status**						
Married	223 (58.5)	2 (0.9)	1			
Single	144 (37.8)	3 (2.1)	2.35 (0.39,14.25)	0.352	1.59 (0.15,16..69)	0.699
Widowed & Divorced	14 (3.7)	1 (7.1)	8.50 (0.72,99.97)	0.089*	2.93 (0.17,51.19)	0.461
**Hand washing practice after latrine**						
Yes	249 (65.4)	1 (0.4)	1		1	
No	132 (34.6)	5 (3.8)	9.76 (1.13,84.47)	0.038*	7.52 (0.77,73.89)	0.083
**Washing of vegetables or fruits before eating**						
Yes	228 (59.8)	2 (0.9)	1		1	
No	153 (40.2)	4 (2.6)	3.03 (0.55,16.77)	0.203*	1.49 (0.23,9.87)	0.675

**NB:** *Candidate variable for multivariate analysis at *P*< 0.25 **variable significant at *P*< 0.05 COR**:** crude odds ratio, **AOR:** adjusted odds ratio, **CI:** confidence interval.

In bivariate analysis, patient without hand washing practice after latrine were 9.76 times (COR 9.76: 95% CI: 1.13, 84.47, *p*=0.038) more likely to had *S.typhi* infection when compared with their counter parts. Study participants who did not washing of vegetables or fruits before eating were 3 times (COR: 95% CI: 0.55, 16.77, *p*=0.203) more likely to be infected with *S.typhi* infection even though, not statically significant.

In further analysis, after adjustment for those significantly associated variables using multivariable logistic regression analysis, the association between *S.typhi* infection and age,,marital status, handwashing practice after latrine and washing of vegetables or fruits before eating did not remain statistically significant (AOR 7.52; 95% CI 0.77–73.89, *P =* 0.083), (AOR 1.49; CI 0.23,9.87, *P*=0.675) respectively. In multivariable analysis, patients who came from rural area had 8 times (AOR 8.27: 95% CI: 1.33, 51.55) more likely to had *S.typhi* infection when compared with urban dwellers.

### Antibiotic susceptibility pattern

**Fig 1:**
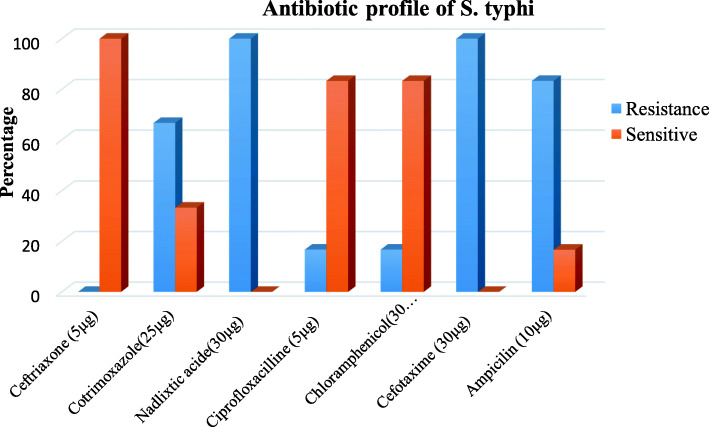
Antibiotic profile of S. typhi

The susceptibility pattern of *S.typhi* isolated from blood culture against seven antimicrobial agent are presented in Fig. 1.The microbial susceptibility testing revealed that all 6(100%) of *S.typhi* isolates showed sensitivity to Ceftriaxone. All 6(100%) isolates showed resistant to Nalidixic acid and Cefotaxime and 5(83.3%) susceptible to Chloramphenicol and Ciprofloxaciline. Resistance to ampicillin was observed 83.3% of *S.typhi* MDR (resistance to three or more antibiotics) was observed among 83.3% (5 of 6) of *S.typhi* isolates (Table 4). The overall resistance for different antibiotics was ranged from 0 to 100%.

**Table 4:** Multidrug resistance patterns among *S.typhi* isolated from patient with febrile illness infection at Adarea Hospital, Southern Ethiopia, and 2018.Ethiopia

Resistant types	Resistance isolates No. (%)
NA, CF, AMP	2 (66.7%)
NA, CF, AMP,CPR	1 (16.7%)
NA, CF, AMP,COT	1 (16.7%)
NA, CF, AMP, COT,CHL	1 (16.7%)

**NA= Nalidixic acid, CF=Cefotaxime, AMP= Ampicillin, CPR= Ciprofloxaciline, COT=Cotrimoxazole, CHL=Chloramphenicol.**

## Discussion

In this study, the prevalence of *S*.typhi among febrile illness patients at Adare General Hospital was 1.6% [95% confidence interval (CI): 0.5–2.9]. This finding was lower than study conducted in Shashemene Ethiopia 5% [15], Central Ethiopia (4.1%) [16], in Indonesia (15.5%) [17] and Lalitpur 4.1% [18]. Similar findings were also reported in India 2.5% [19] and Nepal 1.2% [20]. This difference might be due to geographic setting of the study district, the disparity in study population, time of the studies. Moreover, mode of the laboratory investigation technique disparity also have an effect on the result.

Regarding residence of study participants, it had significant association with *S.typhi* infection where patients living in rural area had 8 times higher risk of having *S.typhi* compared to those who live in urban area. This might be due to lack of access to safe water and hygienic edification, lack of toilet and/or hand washing exercise after toilet, open defecation practices near to the springs and rivers, insufficient medical care, low socio-economic status, poor personal hygiene are possible reason [21, 22].

*S.typhi* is one of the eight highly antibiotic resistant bacteria [23]. In our study all or 100 % of the isolates showed resistant to nalidixic acid. This finding was similar to the finding of Bangladesh and Nepal which shows 100 and 92% resistance, respectively to nalidixic acid [18, 24]. This augmentation may be due substandard supply, condensed antimicrobial therapy, medication sharing, fake drugs, bacterial advancement, climate changes and poor-quality drug.

In this study, most of the *S.typhi* isolates showed higher resistance to Ampicillin (83.3). This was similar to the study conducted in Kenya [25] and previous study done in Ethiopia [26] .This could be due to the availability and handling of these drugs from drug shops/pharmacy and lack of understanding in the management of antimicrobials.

The finding of this study shows all isolate of *S.typhi* were sensitive to ceftriaxone. Similar finding was reported in study done in Bangladesh and Lalitpure, Nepal which shows 100% sensitive to ceftriaxone [18, 23]. In this study chloramphenicol susceptible to *S.typhi* was observed, 83.3%. This finding was similar with study done in India which shows 87.4% of *S.typhi* was sensitive to Chloramphenicol [27].

Additionally, the episodes of S.typhi isolates resistant for more than two drugs were high (83.3%). The enhancement of this is possibly due to mobile genetic units (including plasmids, gene cassettes in integrons and transposons) [28], inadequate access to effective drugs, and abridged antimicrobial therapy [28, 29].

### Limitations

The study was limited on small sample size, sensitivity /specificity and type of sample like stool.

## Conclusion

Bacteraemeia with S.typhi was isolated in 6/379 (1.6%) of febrile patients in our study population. It is an important differential in unwell patients presenting to our hospital and should be considered alongside other bacteraemias seen in our setting, such as *Staphylococcus aureus* (seen in 25 patients, 6.56%), Enterococci (3 patients, 0.8%), and other gram negative organisms (E.coli, proteus, klebsiella, pseudomonas 8 patients, 2.1%.) This study confirms patients in our setting who are unwell with suspected typhoid fever should be treated empirically with Ceftriaxone intravenously. When available, antimicrobial sensitivity results should be used to guide decisions regarding oral antibiotic follow-on options (such as the use of cotrimazole or ciprofloxacin.) Ongoing surveillance is needed in Ethiopia to monitor changes in susceptibility patterns and to guide empirical treatment choices, to strengthen our antibiotic stewardship programmes and combat the rise of antimicrobial resistant pathogens.

## Supplementary Information

**Additional file 1.**

## Data Availability

The datasets used and analyzed during the current study available from the corresponding author on reasonable request.
