# Supplementary material for: Prevalence of Salmonella enterica serovar Typhi infection, its associated factors and antimicrobial susceptibility patterns among febrile patients at Adare general hospital, Hawassa, southern Ethiopia
Source: BMC Infect Dis. 2021 Jan 7;21:30. doi: 10.1186/s12879-020-05726-9 (PMC7791979; doi:10.1186/s12879-020-05726-9)
Supplement: Supplementary file 1 — Additional file 1. [file 12879_2020_5726_MOESM1_ESM.docx]

**Prevalence of *Salmonella entericaserovar*typhi infection and its associated factors and antimicrobial susceptibility patterns among febrile patients attending Adare General Hospital Hawassa, Southern Ethiopia**

**Roza Nasir Awol ^*1^, Dawit Yihdego Reda^2^, Deresse Daka Gidebo^3^**

**E-mails of Authors**

*^1^ Correspondence Author

^1^ [rozanasir796@gmail.com](mailto:rozanasir796@gmail.com), ^2^[dawitbgm@yahoo.com](mailto:dawitbgm@yahoo.com) , ^3^[drsdk200@gmail.com](mailto:drsdk200@gmail.com)

**ENGLISH VERSION QUESTIONNAIRE**

**Part 1: Socio-demographic data and history of patients**

| **S.No** | **Variables** |  |
| --- | --- | --- |
| 1. | Code |  |
| 2. | Age |  |
| 3. | Sex | 1. M 2. F |
| 4 | Place of residence | 1. Urban 2. Rural |
| 5 | Religion | 1. Muslim 2. orthodox 3. protestant 4. other specify ____________ |
| 6. | Ethnicity | 1. Sidama 2. Wolaita 3. Gurage 4. other specify ______________ |
| 7. | Family size | _____ |
| 8. | Marital status | 1. Married 2. single 3. divorced 4. widowed |
| 9 | Occupation | 1. Merchant 2. Governmental employment 3. student 4. others specify __________ |
| 10. | Educational status: | 1. Illiterate 2. can read and write 3. 1-8 4. 9-12 5. diploma and above |
| 11. | Where do you get drinking water? | 1. / river 2. well water 3. unprotected spring water 4. tap water |
| 12. | Do you treat water for drinking? | 1. Yes 2. No |
| 13. | If the answer is yes for Q13How do you treat water for drinking? | 1. Boil 2. Sieve 3. decanting 4. Any other (Specify) |
| 14. | Do you have Latrine? | 1. Yes 2. No |
| 15. | If yes for Q15, is functional? | 1. Yes 2. Not |
| 16. | If no for Q15, where do you defecate? | 1. Directly excrete into the pond 2. directly excrete on the ground 3. Other___________ |
| 17. | Do you wash your hand after latrine? | 1. Yes 2. No |
| 18. | If Q18 answer yes, how often do you use soap while washing your hands | 1. always 2. sometimes 3. Never |
| 19. | Do you wash your hands before preparing food | 1. Yes 2. No |
| 20. | When do you wash your hands? | 1. before meal 2. after meal 3. before meal &after meal 4. Not at all |
| 21. | Where do you eat your meal? | 1. Hotel only 2. Home only 3. Home and hotel |
| 22. | Did you Eat food from street vendor | 1. Yes 2. No |
| 23. | Do you wash vegetables/fruit before eating? | 1. yes 2. No |
| 24. | Recent infection with typhoid fever in the family members | 1. Yes 2. No |
| 25. | Have you ever suffered from typhoid fever? | 1. Yes 2. No |

Laboratory forms

For laboratory use only

|  | Code no |  |
| --- | --- | --- |
|  | Presumptive test of *S*.typhi on selective media | 1. Positive 2. negative |
| Identification steps for suspective colonies | | |
| 1 | Glucose and lactose fermentation and H_2_S production |  |
| 2 | Lysine decarboxylase |  |
| 3 | SIM test |  |
| 4 | . Citrate utilization test |  |
| 5 | Urease test |  |
| Antimicrobial susceptibility result (Resistance (R), Intermediate (I) or sensitive(S) | | |
| 1. | Ciprofloxacin (5μg) |  |
| 2. | Cotrimoxazole (25μg) |  |
| 3. | Chloramphenicol (10μg) |  |
| 4. | Ceftrazone (5μg) |  |
| 5. | Cefotaximine |  |
| 6. | Ampicillin (30μg) |  |
| 7. | Naldistic acid |  |

Sig. ____________ Date______________
